# The interactive effect of pre-pregnancy overweight and obesity and hypertensive disorders of pregnancy on the weight status in infancy

**DOI:** 10.1038/s41598-019-52140-6

**Published:** 2019-11-04

**Authors:** Jiahong Sun, Hong Mei, Shuixian Xie, Lisha Wu, Yulong Wang, Wenhua Mei, Jianduan Zhang

**Affiliations:** 1 Department of Maternal and Child Health, School of Public Health, Tongji Medical College, Huazhong University of Science and Technology, 13 Hangkong Rd., Wuhan, 430030 Hubei China; 2 Wuhan Children’s Hospital, Tongji Medical College, Huazhong University of Science and Technology, 100 Hongkong Rd., Wuhan, 430016 Hubei China; 3 Department of Information, Zhuhai Public Hospital Authority, 351 East Meihua Rd., Zhuhai, 519000 Guangdong China; 4 Department of Epidemiology, Jinan University, 601 Huangpuxi Rd., Guangzhou, 510632 Guangdong China

**Keywords:** Risk factors, Epidemiology

## Abstract

We aimed to assess whether hypertensive disorders of pregnancy (HDP) could modify the effect of pre-pregnancy overweight or obesity (OWO) on the risk of offspring high body mass index (BMI) in infancy. A total of 3,765 mother-child pairs were recruited from two Chinese birth cohorts. BMI ≥ 85^th^ percentile, based on World Health Organization criteria, was defined as a high BMI for the risk of developing severe obesity in later life. Logistic regression analysis was used to assess the combined effects and multiplicative interactions of pre-pregnancy OWO + HDP on offspring high BMI. Relative excess risk due to interaction (RERI) or attributable proportion (AP) was used to estimate additive interactions. RERI > 0 or AP > 0 indicates a significant additive interaction. Compared with the non-OWO and normal blood pressure group, the combination of OWO + HDP was positively associated with offspring high BMI at 12 months of age [OR 3.10 (95%CI 1.59, 6.04)], with 51% of the effects attributed to an additive interaction [AP 0.51 (95%CI 0.13, 0.89)]. An interactive effect was found between the pre-pregnancy OWO + HDP and offspring high BMI in infancy. Interventions to control pre-pregnancy OWO and HDP are important to prevent obesity and associated adverse outcomes in offspring.

## Introduction

The prevalence of overweight or obesity (OWO) in both children and adults has been increasing substantially worldwide^1–3^. OWO that develops in childhood increases the early risk of cardiovascular damage resulting in an increased incidence of cardiovascular morbidity and mortality in adulthood^4^.

To reduce the incidence of obesity and associated adverse outcomes, exposure to potential risk factors early in life that contribute to a high body mass index (BMI) status should first be considered. There is an increasing interest in understanding the role of the intrauterine environment on the development of obesity in offspring, e.g., maternal pre-pregnancy weight, gestational weight gain and blood pressure (BP) during pregnancy^5,6^, etc. Pre-pregnancy overweight status is considered as a risk factor for the high BMI status in offspring by influencing the intrauterine environment. However, results have not been consistent. For instance, McCloskey *et al*. reported that high maternal pre-pregnancy BMI was a risk factor for obesity in newborn offspring^7^. Yet others reported no association between pre-pregnancy high BMI and offspring OWO at 1 year of age^8^. These inconsistent findings suggest that other variables influencing the intrauterine environment might be modifying the association between maternal BMI and BMI status in offspring. A number of studies have found that hypertensive disorders of pregnancy (HDP) could also increase the risk of offspring obesity, which might be partly attributed to a change in the intrauterine environment^9,10^.

Therefore, in this study, we aimed to investigate the independent and combined effects of pre-pregnancy OWO and HDP on the risk of offspring high BMI status in infancy and explore the related additive and multiplicative interactions further.

## Subjects and Methods

### Study participants

Data were extracted from two Chinese birth cohorts. The first birth cohort (Cohort A) was conducted in the cities of Shenyang, Wuhan, and Guangzhou from April 2009 to March 2010. The second cohort (Cohort B) was performed in Zhuhai city from May 2014 to December 2016. In Cohort A, maternal age, infant sex, birthdate, delivery mode, gestational weight gain (GWG), BP measurements during pregnancy and gestational condition (e.g. taking antihypertensive medications, present of diabetes mellitus, etc.) were obtained from the patient Perinatal Health Booklets; maternal height and infant weight and length were measured by trained nurses; and maternal pre-pregnancy weight, gestational age, household income, educational level, maternal secondhand smoke exposure during pregnancy, the feeding practices during the first month of life, and cities were obtained using self-reported questionnaires. In Cohort B, maternal age, infant sex, birthdate, delivery mode, BP measurements during pregnancy, and gestational conditions were obtained from the Maternal and Child Health Information System; infant weight and length were measured by trained nurses; and maternal pre-pregnancy weight and height, gestational age, household income, educational level, maternal secondhand smoke exposure during pregnancy, the feeding practices during the first month of life, and GWG were obtained from self-reported questionnaires.

**Figure 1:**
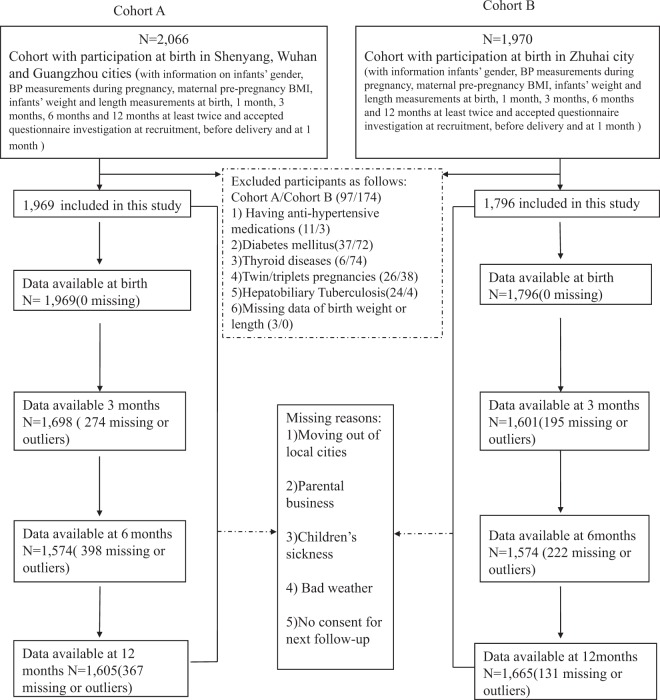
Flowchart of participants. Cohort A was conducted in Shenyang, Wuhan and Guangzhou cities (2009–2010). Cohort B was conducted in Zhuhai city (2014–2016).

A total of 4,036 mother-child pairs (2,066 in Cohort A and 1,970 in Cohort B) were originally recruited with information on maternal pre-pregnancy weight and height, BP measurements during pregnancy, infant sex, infant weight and length measured at least twice at birth, 1, 3, 6 and 12 months, and questionnaires were filled out during the study recruitment, before delivery, and 1 month after giving birth. Among all participants, 97 mother-child pairs in Cohort A and 174 mother-child pairs in Cohort B were excluded for mothers using antihypertensive medications, having diabetes mellitus, tuberculosis, thyroid or hepatobiliary diseases, or a non-singleton pregnancy, or missing data in infants’ weight and length at birth. A total of 3,765 mother-child pairs (1,969, 1,698, 1,574, and 1,605 participants in Cohort A, and 1,796, 1,601, 1,574 and 1,665 in Cohort B with available data at birth, 3, 6 and 12 months, respectively) were finally included in this analysis. No significant differences were observed in the main variables between the samples used for analysis and those originally included (Supplementary Table S1). Therefore, the possible withdrawal bias was eliminated in this study. A flowchart of the study design is presented in Fig. 1.

All protocol-required procedures in the four cities of Cohort A and B were carried out in accordance with the Declaration of Helsinki and data were collected by trained staff. The ethical approval was obtained from the Ethics Committees of the Tongji Medical College, Huazhong University of Science and Technology, and all participants signed informed consent before study participation.

### Anthropometric assessments

In Cohort A, infant weight and length at birth, 3, 6, and 12 months were measured twice by trained health staff with an electronic scale (WHS-I, Wuhan Computer Software Development) to the nearest 0.05 kg and a length measurement instrument (WHS-I) to the nearest 0.1 cm, respectively. In Cohort B, infant weight and length at the corresponding months were measured twice by trained health staff with an electronic weighing scale (Hengxin HCS-20-YE) and a horizontal baby bed (Hengxin HX-II), respectively. For all measurements, average values were used. The weight and length at each visit were linearly adjusted by the measured intervals to the exact duration of the corresponding months based on World Health Organization criteria^11^. For instance, an infant that weighed 3.5 kg at birth and 11.0 kg at the 370-day measurement had an adjusted 12-month weight of (11.0–3.5 kg) × (365.25/370) + 3.5 = 10.9 kg (12-month exact days were: 12 × 30.4375 = 365.25days). Maternal height in Cohort B was measured by trained staff using a stadiometer (Leicester height measure; Invicta Plastic, Leicester, UK).

### Covariates

Births were divided into vaginal and cesarean deliveries. Infant birth weight and length, gestational age, maternal age, and maternal GWG were continuous variables. GWG was defined as the maternal weight before delivery minus their weight before pregnancy. The maternal educational level was classified as primary and secondary school, high school, university/college, and postgraduate education. Secondhand smoke exposure during pregnancy was defined for non-smokers as being in the presence of cigarette smoking for at least 15 minutes per day or at least 1 day per week^12^. Feeding patterns at 1 month were classified as exclusive breastfeeding (WHO definition^13^), mixed feeding (both formula and breast milk), and formula feeding (exclusively formula without breast milk). Household income was categorized as <3000RMB ($426.6), 3001–5000RMB ($426.7–711.0) and 5001–8000RMB ($711.1–1137.6), or ≥8001RMB ($1137.7). The adjusted models were performed with the mentioned confounders and cohort variable adjusted. The missing data was compensated using a non-parametric missing value imputation (missForest based on random forest package in R3.5.2)^14^. The included data and the imputed datasets representing the main variables did not show significant differences (Supplementary Table S2).

### Definition of maternal pre-pregnancy OWO and HDP

BMI was calculated by dividing weight in kilograms by height in meters squared. An expert panel of the World Health Organization appealed for additional research on the health implications of infants using a BMI indicator based on length^15^. It has been shown that the 85^th^ percentile cutoff of BMI in infancy defined a highly-specific threshold for an increased risk of becoming overweight or obesity in early childhood^16,17^. In addition, compared with weight for length, BMI was recognized as a better indicator of body adiposity in infancy^18^. To make the data internationally comparable and able to be tracked over time, we defined a BMI ≥ 85^th^ percentile as a high BMI status at 12 months of age, according to the BMI percentiles of the WHO Child-Growth Standards^19^. The maternal pre-pregnancy BMI status was classified as non-OWO (BMI < 24 kg/m^2^) and OWO (BMI ≥ 24 kg/m^2^), according to the Working Group on Obesity in China^20^. Gestational BP status was divided into the HDP (systolic BP ≥ 140 mmHg and/or diastolic BP ≥ 90 mmHg during pregnancy) and normal BP (NBP, systolic BP/diastolic BP < 140/90 mmHg) groups. We were unable to distinguish specific gestational hypertensive disorders, such as gestational hypertension and preeclampsia since urine protein data were not available. Therefore, the definition of HDP in this study represents a combination of gestational hypertensive disorders during pregnancy, which was similarly defined in a previous study^10^.

### Statistical analysis

All data analyses were conducted using SPSS software, version 16.0 (SPSS, Inc., Chicago, IL, USA). Maternal age, GWG and gestational age (continuous variables) with non-normal distributions were expressed as the median and inter-quartile range (IQR). Maternal education level, feeding patterns at 1 month, mode of delivery, and maternal secondhand smoke exposure during pregnancy (category variables) were presented as N (%). The Kruskal-Wills test was used to compare continuous variables, and the Chi-square test was used to compare categorical variables between the groups. The generalized estimating equation models were used to assess the association of pre-pregnancy BMI and BP during pregnancy with the BMI-z at birth, 3, 6, and 12 months. Both multiplicative (statistical interactions) and additive (biological interaction) interactions^21^ were assessed in this study. A total of three logistic regression models were used to evaluate the effects of pre-pregnancy BMI status and gestational BP status and their multiplicative interactions on the high BMI status of the offspring at 12 months of age, adjusting for potential confounders. The three models are Model 1, the unadjusted model; Model 2, adjusted for birth-related variables (infant sex, gestational age, birth weight and length, and delivery mode), maternal variables (maternal age and GWG) and cohort variable and; Model 3, a model similar to Model 2 with further adjustments for family environment-related variables (maternal educational level, maternal secondhand smoke exposure during pregnancy, household income, and feeding patterns at 1 month). These potential variables were included because there were either well-established associations with the offspring BMI-z values or because there was at least a 10% variation in the interest estimates if removed^22^. *P-*values <0.05 were considered statistically significant.

The odds ratios (OR) of OR_00_, OR_10_, OR_01,_ and OR_11_ were calculated for the Non-OWO + NBP (reference group), Non-OWO + HDP, OWO + NBP and OWO + HDP groups, respectively. The calculation of the relative excess risk of interaction (RERI = OR_11_ − OR_10_ − OR_01_ + 1), attributable proportion (AP = RERI/OR_11_) and the 95% confidence interval(CI) was performed using a Microsoft Excel spreadsheet generated by Andersson *et al*.^23^. RERI > 0 or AP > 0 indicates a biologically additive interaction, which had been widely used in previous studies^24,25^.

## Results

### Subject characteristics

Table 1 shows the characteristics of mothers and infants based on the combined maternal pre-pregnancy BMI status and BP status during pregnancy. OWO + HDP mothers had the highest percentage of offspring with a high BMI status at 12 months of age (56.8%). Non-OWO + HDP mothers had the highest weight gain during pregnancy [median (IQR): 16.0 (12.3, 20.0) kg]. OWO + HDP mothers were more on average older [30.0 (27.0, 32.0) years], having higher pre-pregnancy BMI [25.4 (24.7, 27.3) kg/m^2^], more likely to live in low income household (46.7%), and have a higher proportion of infants with low birth weight (2.2%). Gestational age, maternal secondhand smoke exposure during pregnancy, maternal educational level, delivery mode, feeding pattern during the first month of life, and infant sex are also presented.

**Table 1:** Parental and infants characteristics stratified by pre-pregnancy BMI status and BP status during pregnancy^†^.

Characteristics	Participants	Non-OWO^‡^		OWO^‡^		*P*-value
	(N)	NBP^‡^ (n = 3,261)	HDP^‡^ (n = 140)	NBP^‡^ (n = 319)	HDP^‡^ (n = 45)	
Maternal age (yrs)	3,765	28.0 (26.0, 30.0)	28.0 (26.0, 30.0)	29.0 (27.0, 32.0)	30.0 (27.0, 32.0)	<0.001
Gestational weight gain (kg)	3,765	15.0 (12.4, 19.0)	16.0 (12.3, 20.0)	13.0 (10.0, 17.1)	15.0 (10.0, 21.0)	<0.001
Gestational age (wks)	3,765	39.0 (38.0, 40.0)	39.0 (38.0, 40.0)	39.0 (38.0, 40.0)	39.0 (38.0, 40.0)	0.301
Pre-pregnancy BMI^‡^ (kg/m^2^)	3,765	19.7 (18.4, 21.3)	20.7 (18.6, 21.6)	25.2 (24.5, 26.7)	25.4 (24.7, 27.3)	<0.001
Birth weight						<0.001
Low	67	62 (1.9)	2 (1.4)	2 (0.6)	1 (2.2)	
Normal	3,503	3,046 (93.4)	134 (95.7)	283 (88.7)	40 (88.9)	
Macrosomia	195	153 (4.7)	4 (2.9)	34 (10.7)	4 (8.9)	
Secondhand smoke exposure during pregnancy						0.459
Yes	943	829 (25.4)	28 (20.0)	76 (23.8)	10 (22.2)	
No	2,822	2,432 (74.6)	112 (80.0)	243 (76.2)	35 (77.8)	
Maternal education						0.419
Primary and secondary school	504	428 (13.1)	20 (14.3)	52 (16.3)	4 (8.9)	
High school	788	681 (20.9)	27 (19.3)	66 (20.7)	14 (31.1)	
University/college	2,267	1,965 (60.3)	86 (61.4)	190 (59.6)	26 (57.8)	
Postgraduate	206	187 (5.7)	7 (5.0)	11 (3.4)	1 (2.2)	
Household income (RMB)						0.013
<3000 ($426.6)	963	810 (24.8)	46 (32.9)	86 (27.0)	21 (46.7)	
3001–5000 ($426.7–711.0)	905	790 (24.2)	31 (22.1)	74 (23.2)	10 (22.2)	
5001–8000 ($711.1–1137.6)	736	657 (20.1)	25 (17.9)	51 (16.0)	3 (6.7)	
≥8001 ($1137.7)	1,161	1,004 (30.8)	38 (27.1)	108 (33.9)	11 (24.4)	
Feeding pattern at 1^st^ month						0.179
Exclusive breastfeeding	1,555	1,369 (42.0)	57 (40.7)	114 (35.7)	15 (33.3)	
Mixed feeding	1,810	1,560 (47.8)	64 (45.7)	162 (50.8)	24 (53.3)	
Formula feeding	400	332 (10.2)	19 (13.6)	43 (13.5)	6 (13.3)	
Delivery						<0.001
Vaginal	1,744	1,560 (47.8)	57 (40.7)	119 (37.3)	8 (17.8)	
Cesarean	2,021	1,701 (52.2)	83 (59.3)	200 (62.7)	37 (82.2)	
Infants gender						0.016
Boys	2,033	1,740 (53.4)	69 (49.3)	195 (61.1)	29 (64.4)	
Girls	1,732	1,521 (46.6)	71 (50.7)	124 (38.9)	16 (35.6)	
BMI status at 12 months		n = 2845	n = 117	n = 271	n = 37	<0.001
Normal BMI status	2,360	2,080 (73.1)	77 (65.8)	187 (69.0)	16 (43.2)	
High BMI status	910	765 (26.9)	40 (34.2)	84 (31.0)	21 (56.8)	

^†^Data are expressed as median and interquartile range (IQR) or N (%). Kruskal-Wills test and Chi-square test were used to compare continuous and categorical variables between groups. ^‡^BMI: body mass index; BP: blood pressure; Non-OWO: non-overweight or obesity; NBP: normal blood pressure; OWO: overweight or obesity; HDP: hypertensive disorders of pregnancy.

### Maternal pre-pregnancy BMI status, BP status during pregnancy, and offspring high BMI

As shown in Table 2, compared with the non-OWO group, pre-pregnancy OWO was independently associated with offspring BMI-z levels at birth [*β* 0.31, 95%CI (0.20, 0.42)] and 12 months of age [*β* 0.14, 95%CI (0.01, 0.27)]. Maternal HDP was independently associated with offspring BMI-z values at 12 months of age [*β* 0.24 (0.07, 0.41)] compared with the NBP group (all *P* < 0.05). Compared with infants from Non-OWO + NBP mothers, the BMI-z values of infants from OWO + HDP mothers were on average 0.62 units greater at 12 months of age (*P* = 0.002).

**Table 2:** Associations of pre-pregnancy BMI status and gestational BP status with repeatedly measured BMI-z at birth, 3, 6 and 12 months^†^.

		Birth (N = 3,765)	*P*-value	3months (N = 3,229)	*P*-value	6months (N = 3,140)	*P*-value	12 months (N = 3,270)	*P*-value
		*β* (95%CI)		*β* (95%CI)		*β* (95%CI)		*β* (95%CI)	
**Independent effect**									
Pre-pregnancy BMI status^‡^									
Non-OWO^‡^		Reference		Reference		Reference		Reference	
OWO^‡^		0.31 (0.20, 0.42)	<0.001	0.10 (−0.02, 0.21)	0.102	0.07 (−0.06, 0.19)	0.287	0.14 (0.01, 0.27)	0.033
**BP status during pregnancy**									
NBP^‡^		Reference		Reference		Reference		Reference	
HDP^‡^		−0.12 (−0.25, 0.02)	0.083	0.14 (−0.03, 0.31)	0.099	0.15 (−0.02, 0.31)	0.075	0.24 (0.07, 0.41)	0.007
**Combined effect**									
Pre-pregnancy BMI status^‡^	Gestational BP status^‡^								
Non-OWO^‡^	NBP^‡^	Reference		Reference		Reference		Reference	
Non-OWO^‡^	HDP^‡^	−0.13 (−0.29, 0.02)	0.093	0.16 (−0.03, 0.35)	0.102	0.12 (−0.07, 0.31)	0.213	0.16 (−0.03, 0.34)	0.095
OWO^‡^	NBP^‡^	0.30 (0.18, 0.43)	<0.001	0.11 (−0.02, 0.23)	0.093	0.06 (−0.08, 0.19)	0.418	0.11 (−0.03, 0.24)	0.124
OWO^‡^	HDP^‡^	0.24 (0.01, 0.46)	0.041	0.19 (−0.14, 0.52)	0.266	0.30 (−0.01, 0.61)	0.055	0.62 (0.22, 1.01)	0.002

^†^Generalized estimating equation models. Adjusted for cohort, birth weight and length (except for BMI-Z at birth) infant sex, gestational age, delivery mode, maternal age, maternal gestational weight gain and educational level, household income, secondhand smoke exposure, feeding patterns at 1 month (Pre-pregnancy BMI status and BP during pregnancy adjusted for each other when assess the independent effect). ^‡^BMI: body mass index; BP: blood pressure; HDP: hypertensive disorders of pregnancy; Non-OWO: non-overweight or obesity; NBP: normal blood pressure; OWO: overweight or obesity.

**Figure 2:**
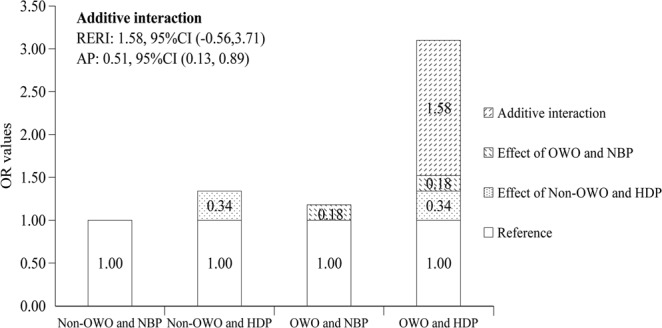
The additive interaction of maternal pre-pregnancy BMI status and BP status during pregnancy on high BMI status in infants at 12 months old in adjusted Model 3. RERI, relative excess risk due to interaction; AP, attributable proportion; Non-OWO: non-overweight or obesity; OWO: overweight or obesity; NBP: normal blood pressure; HDP: hypertensive disorders of pregnancy.

As shown in Table 3, in an unadjusted analysis, OWO + HDP mothers had an increased risk of having offspring with a high BMI status [OR 3.57 (95%CI 1.85, 6.88)] at 12 months of age, compared with Non-OWO + NBP mothers. There was a 54.0% [AP 0.54, 95%CI (0.20, 0.89)] increased risk attributed to additive interactions. The results of Model 2 and Model 3 were similar to those of the unadjusted model. In Model3, after the adjustment for birth-related variables, maternal variables, cohort, and family environment-related variables, OWO + HDP was still also associated with offspring high BMI status at 12 months of age [OR 3.10 (95%CI 1.59, 6.04)]. There was also an additive interaction between OWO + HDP and offspring high BMI status at 12 months [AP 0.51, 95%CI (0.13, 0.89)] (Table 3 and Fig. 2). No multiplicative interactions were observed in the 3 models.

**Table 3:** The interactive effect between maternal pre-pregnancy BMI status and BP status during pregnancy on high BMI status in infants at 12 months old^†^.

Pre-pregnancy BMI status^‡^	BP status during pregnancy^‡^	Model1^†^	*P*-value	Model2^†^	*P*-value	Model3^†^	*P*-value
		OR (95% CI)		OR (95% CI)		OR (95% CI)	
Non-OWO^‡^	NBP^‡^	Reference		Reference		Reference	
Non-OWO^‡^	HDP^‡^	1.41 (0.96, 2.09)	0.083	1.36 (0.92, 2.03)	0.126	1.34 (0.90, 2.00)	0.146
OWO^‡^	NBP^‡^	1.22 (0.93, 1.60)	0.147	1.18 (0.90, 1.56)	0.235	1.18 (0.89, 1.56)	0.240
OWO^‡^	HDP^‡^	3.57 (1.85, 6.88)^*^	<0.001	3.25 (1.67, 6.31)^**^	0.001	3.10 (1.59, 6.04)^**^	0.001
**Multiplicative interaction**							
OWO*HDP^‡^		2.07 (0.93, 4.61)	0.075	2.01 (0.89, 4.53)	0.091	1.95 (0.87, 4.40)	0.107
**Additive interaction**							
OWO*HDP (RERI/AP)^‡^		1.94 (−0.47, 4.34)/0.54 (0.20, 0.89)		1.70 (−0.53, 3.92)/0.52 (0.16, 0.89)		1.58 (−0.56, 3.71)/0.51 (0.13, 0.89)	

^†^Logistic regression analysis. Model1: Unadjusted model; Model2: Adjusted for cohort, infant sex, gestational age, infant birth weight and length, delivery mode, maternal age and maternal gestational weight gain; Model 3: Adjusted for potential variables in Model2 plus maternal education level, household income, secondhand smoke exposure, and feeding patterns at 1 month. ^‡^AP, attributable proportion; RERI, relative excess risk due to interaction; BMI: body mass index; BP: blood pressure; Non-OWO: non-overweight or obesity; OWO: overweight or obesity; NBP: normal blood pressure; HDP: hypertensive disorders of pregnancy.

### Subgroup analysis stratified by cohorts

The comparison of the baseline characteristics between Cohort A and Cohort B were performed and the results are presented in Supplementary Table S3. Although there is a difference in some variables between the two cohorts, the subgroup analysis showed consistent additive interactions of maternal OWO and HDP on high BMI status in offspring at 12 months in both Cohort A [AP 0.50, 95%CI (0.01, 0.99)] and Cohort B [AP 0.60, 95%CI (0.21, 0.99)] (Supplementary Table S4), which are similar to the results of the pooled analysis.

## Discussion

Our findings indicated that maternal pre-pregnancy OWO and HDP were independent risk factors for offspring high BMI-z values in infancy. A significant combined effect of OWO and HDP on high BMI-z values of the infants was observed at 12 months of age. We also found that the OWO + HDP in mothers synergistically increased the risk of having offspring with a high BMI status at 12 months compared with Non-OWO + NBP mothers. A total of 51.0% of the increased risk was attributable to additive interactions.

Previous studies have inconsistently reported the effects of maternal pre-pregnancy OWO on offspring high BMI status. Offspring of mothers, who had a BMI ≥ 30 kg/m^2^ during pre-pregnancy, had a higher risk of being obese at 2 to 4 years of age^26^. However, Lindberg *et al*. reported pre-pregnancy OWO was not a risk factor for offspring OWO at 1 year old of age^8^. In this study, we showed that maternal pre-pregnancy OWO was associated with higher offspring BMI-z values at 12 months of age, which suggests that the effects of pre-pregnancy OWO on offspring high BMI could emerge early in life. The discrepancy between the findings of Lindberg *et al*. and ours might be due to the different ethnic groups studied (American Indian children *vs*. Chinese children), the potential factors considered (unmentioned *vs*. infant-related variables, maternal variables, and family-related variables), sample sizes (471 *vs*. 3,270), classification references (CDC growth charts for the United States *vs*. WHO Child-Growth Standards), as well as other factors.

To our knowledge, most previous research has primarily focused on preeclampsia. For example, a Swedish research group showed that preeclampsia during pregnancy had a 1.54- and 1.40-fold increased risk of having offspring that became overweight (BMI ≥ 25 kg/m^2^) or obese (BMI ≥ 30 kg/m^2^) in early middle age^27^. A meta-analysis demonstrated that mothers with preeclampsia during pregnancy had an increased risk of having children with higher BMI at ≥10 years of age, but not in children <10 years of age^28^. In the present study, we found that mothers with HDP had an independently increased risk of having offspring with higher BMI-z values as early as 12 months of age. These different findings could be explained by the diverse backgrounds of the study populations, the various ages of the measured outcomes, and the different definitions of the hypertensive disorders, etc. The findings similar to our results have also demonstrated a positive association of HDP with high BMI-z in offspring during childhood^29^. A birth cohort in Jiaxing, China showed that HDP was a risk factor for obesity in 4–7 year-old children^10^. However, the results were based on one small city in China, and some potential confounders such as gestational weight gain were not considered. Based on the data from four representative cities in China, our results showed a significant association between maternal HDP and high infant BMI-z scores, which suggests the importance of early detection of HDP.

So far, limited studies have assessed the combined effects of maternal pre-pregnancy high BMI status and elevated BP status during pregnancy on offspring high BMI status. In the present study, the maternal OWO + HDP had positive associations with the offspring BMI-z values at 12 months. We provided evidence that OWO + HDP in mothers synergistically increased the risk of offspring high BMI status at 12 months compared with non-OWO + NBP mothers. Moreover, 51.0% of the effects were attributed to an additive interaction, which means that the estimated combined effect of maternal OWO and HDP, on an additive scale, was greater than the sum of the two sole effects. Given these results, it seems plausible that HDP modifies the association between maternal pre-pregnancy OWO and high BMI status in offspring. Therefore, in addition to maintaining healthy weight status during pre-pregnancy, it is important that BP is monitored during pregnancy to attenuate the risk of having offspring with a high BMI status.

Direct intrauterine mechanisms could partly explain the potential effects of maternal pre-pregnancy OWO, HDP, and the interactions of these two disorders^30,31^. There are several possible mechanisms regarding the association between pre-pregnancy obesity and high offspring BMI status. First, adipose tissue function in offspring could be increased by a maternal obesity program that causes alterations in signaling and adipocyte morphology^5^. For instance, maternal pre-pregnancy obesity increases circulation of glucose, lipids, leptin, and others. These factors could be transferred to the fetus through the placenta and could contribute to the epigenetic programming (e.g. adiponectin and adipocytokine gene expression) of offspring, which has been linked to OWO in offspring^32–34^. Second, maternal OWO could also affect the orexigenic factors (NPY) in offspring appetite control systems by altering the hypothalamic architecture, which leads to overeating and subsequent rapid weight gain^35^. Third, mothers with unhealthy eating habits and pre-pregnancy obesity could cause offspring to have increased energy intakes and BMI as well through unhealthy feeding practice^36,37^. Although the mechanisms between HDP and high BMI in offspring is not clear, several possible mechanisms have been proposed. First, altered adipose tissue gene (e.g. leptin gene) expression in the placenta of pregnant women with preeclampsia^38–40^ might influence offspring body weight^41–43^. Second, HDP was shown to be related to oxidative stress in offspring, which was associated with obesity^44,45^. In general, both OWO and HDP in mothers might impair fetal growth through a changed intrauterine nutrition environment^5,6,46,47^, which could synergistically alter adipose tissue morphology and metabolism, modify the hormones and epigenome, and regulate appetite pathways^48,49^. Thus, through these mechanisms, maternal obesity and HDP might jointly increase the risk of high BMI status in offspring.

We used pooled data from the two birth cohorts in four cities located in Northern, Central and Southern China that were more representative of Chinese populations, thus increasing the external validity of our results. However, several limitations should also be mentioned. First, since urine protein concentrations were unavailable, we were unable to differentiate the different types of hypertensive disorders, such as chronic hypertension, gestational hypertension or preeclampsia, and therefore, could not determine which disorders would be better predictors of offspring obesity than a BP ≥ 140/90 mmHg. Second, we did not consider BP status at different trimesters because only a limited number of participants received BP measurements at all trimesters. The prevalence of HDP might have been overestimated; however, the primary aim of this study was not to make a clinical diagnosis of hypertension but to assess the interactive effects of maternal pre-pregnancy OWO and HDP in a general population. Third, to improve statistical power, we combined overweight and obese women during pre-pregnancy into one group. Larger populations with more detailed categories of maternal pre-pregnancy weight status and BP measurements during different trimesters are needed for future studies. Fourth, maternal pre-pregnancy weight in both cohorts was self-reported. However, the self-reported weight has been demonstrated to be reliable and valid in epidemiologic studies^50^. Fifth, there are 21 individuals with a BMI ≥ 85^th^ percentile at 12 months, which limits our ability to assess the effect of maternal OWO + HDP on a higher BMI status (e.g. BMI ≥ 90^th^ percentile) in infancy. In this study, we used a BMI ≥ 85^th^ percentile, which has been demonstrated as a cut-off for predicting obesity in early childhood^16,17^. Finally, we only adjusted feeding patterns during the first month because feeding practices were not provided at 3 months or longer in Cohort A. Further studies are needed to consider feeding practices for a longer period.

## Conclusions

In conclusion, there was an additive interaction of maternal pre-pregnancy OWO and HDP on offspring high BMI status. Therefore, monitoring maternal weight status before pregnancy and measuring BP during pregnancy has important implications to identify high-risk infant offspring for the early prevention of a high BMI status to protect them from health risks in later life.

## Supplementary information

Supplementary Table S1–4

## Data Availability

Data are available from authors can be contacted at the Department of Maternal and Child Health Care, School of Public Health, Tongji Medical College, Huazhong University of Science and Technology, 13 Hangkong Rd., Wuhan, 430030, Hubei, China. Contact information: Jianduan Zhang, jd_zh@hotmail.com or Jiahong Sun, jiahongsun1990@sina.com.
