# Supplementary material for: The interactive effect of pre-pregnancy overweight and obesity and hypertensive disorders of pregnancy on the weight status in infancy
Source: Sci Rep. 2019 Nov 4;9:15960. doi: 10.1038/s41598-019-52140-6 (PMC6828655; doi:10.1038/s41598-019-52140-6)
Supplement: Supplementary file 1 — Supplementary Table S1–4 [file 41598_2019_52140_MOESM1_ESM.pdf]

The interactive effect of pre-pregnancy overweight and obesity and hypertensive disorders of pregnancy on the weight status in infancy

Authors

Jiahong Sun[1], Hong Mei[1,2], Shuixian Xie[1], Lisha Wu[1], Yulong Wang[1], Wenhua Mei[3, 4*], Jianduan Zhang[1*]

Affiliation

[1] Department of Maternal and Child Health, School of Public Health, Tongji Medical College, Huazhong University of Science and Technology, 13 Hangkong Rd., Wuhan, 430030, Hubei, China.

[2]Wuhan Children's Hospital, Tongji Medical College, Huazhong University of Science and Technology, 100 Hongkong Rd.,Wuhan,430016, Hubei, China.

[3] Department of Information, Zhuhai Public Hospital Authority, 351 East Meihua Rd., Zhuhai, 519000, Guangdong, China.

[4] Department of Epidemiology, Jinan University, 601 Huangpuxi Rd., Guangzhou, 510632, Guangdong, China.

[*] Correspondence and request for materials should be addressed to J.Z. (Tel/Fax: +86-83692755; email: jd_zh@hotmail.com and whmei2001@aliyun.com)

Supplementary Table S1. Comparison of characteristics between infant with included data in this study and those originally recruited[†]

	Original data		Included data		*P*
	N	Median(IQR)/ Mean±SE	N	Median(IQR)/ Mean±SE	
Maternal age(yrs)	3919	28.0(26.0, 31.0)	3655	28.0(26.0, 31.0)	0.367
Gestational weight gain(kg)	3966	15.0(12.0, 19.0)	3739	15.0(12.0, 19.0)	0.724
Gestational age (wks)	3959	39.0(38.0, 40.0)	3689	39.0(38.0, 40.0)	0.195
Pre-pregnant weight (kg)	4036	52.0(48.0,57.0)	3765	52.0(48.0,57.0)	0.733
BMI-z score at birth	4033	-0.29±0.02	3765	-0.28±0.02	0.451
BMI-z score at 3 months	3489	0.26±0.02	3299	0.26±0.02	0.928
BMI-z score at 6 months	3328	0.31±0.02	3140	0.31±0.02	0.909
BMI-z score at 12 months	3404	0.49±0.02	3270	0.50±0.02	0.910
		%		%	
Secondhand smoke exposure during pregnancy					0.960
Yes	970	25.7	904	25.6	
No	2807	74.3	2623	74.4	
Maternal education					0.985
Primary and secondary school	498	13.3	475	13.6	
High school	811	21.7	761	21.8	
University/college	2227	59.7	2070	59.4	
Postgraduate	195	5.2	180	5.2	
Household income(RMB)					0.679
<3000	916	25.7	879	26.4	
3001-5000	885	24.8	840	25.2	

5001-8000	719	20.1	674	20.2	
≥8001	1051	29.4	937	28.1	
Feeding pattern at 1st month					0.799
Exclusive breastfeeding	1452	40.5	1408	41.3	
Mixed feeding	1730	48.2	1624	47.6	
Formula feeding	405	11.3	380	11.1	
Delivery mode					0.621
Vaginal	1847	45.8	1744	46.3	
Cesarean	2189	54.2	2021	53.7	
Infant sex					0.988
Boys	2180	54.0	2033	54.0	
Girls	1856	46.0	1732	46.0	

[†]Data are expressed as median and interquartile range (IQR), Mean± standard error (SE) or %. Kruskal-Wills test and t-test were used to compare continuous variables and Chi-square test was used to compare categorical variables between groups. "*P* <0.05" is considered as significant difference.

Supplementary Table S2. Comparison of main variables between included data and imputed data[†]

	Missing	Included data Median(IQR)/%	Imputed data Median(IQR)/%	*P*
Maternal age(yrs)	110	28.0(26.0, 31.0)	28.0(26.0,30.0)	0.853
Gestational age (wks)	76	39.0(38.0, 40.0)	39.0(38.0, 40.0)	0.955
Gestational weight gain(kg)	26	15.0(12.0, 19.0)	15.0(12.0, 19.0)	0.984
Secondhand smoke exposure during pregnancy				0.566
Yes	39	25.6	25.0	
No	199	74.4	75.0	
Maternal education				0.730
Primary and secondary school	29	13.6	13.4	
High school	27	21.8	20.9	
University/college	197	59.4	60.2	
Postgraduate	26	5.2	5.5	
Feeding pattern at 1st month				0.771
Exclusive breastfeeding	147	41.3	41.3	
Mixed feeding	186	47.6	48.1	
Formula feeding	20	11.1	10.6	
Monthly household income (RMB)				0.100
<3000	84	26.4	25.6	
3001-5000	65	25.2	24.0	
5001-8000	62	20.2	19.5	
≥8001	224	28.1	30.8	

[†]Data are expressed as median and interquartile range (IQR) or %. Kruskal-Wills test and Chi-square test were used to compare continuous and categorical variables between groups. "*P* <0.05" is considered as significant difference.

Supplementary Table S3. Comparison of baseline characteristics between Cohort A and B[†]

	Cohort A[‡]	Cohort B[‡]	P
	Median(IQR)/%	Median(IQR)/%	
Maternal age(yrs)	28.0(26.0,31.0)	28.0(26.0,30.0)	0.524
Pre-pregnancy BMI (kg/m^2)	20.0(18.6,21.8)	20.1(18.6,22.1)	0.411
Gestational age (wks)	39.0(38.0,40.0)	39.0(38.0,40.0)	<0.001
Gestational weight gain(kg)	17.0(14.0,20.0)	14.0(11.0,17.0)	<0.001
Birth weight			<0.001
Low	1.1	2.6	
Normal	91.8	94.4	
Macrosomia	7.2	3.0	
Secondhand smoke exposure during pregnancy			<0.001
Yes	19.8	30.8	
No	80.2	69.2	
Maternal education			<0.001
Primary and secondary school	19.8	6.3	
High school	25.8	15.6	
University/college	50.5	70.8	
Postgraduate	3.9	7.2	
Monthly household income (RMB)			<0.001
<3000	42.2	7.4	
3001-5000	33.2	14.0	
5001-8000	16.7	22.7	
≥8001	8.0	55.9	
Feeding pattern at 1st month			<0.001
Exclusive breastfeeding	37.6	45.3	
Mixed feeding	49.3	46.8	
Formula feeding	13.1	7.9	
Delivery			<0.001
Vaginal	32.4	61.6	
Cesarean	67.6	38.4	
Infants gender			0.065
Boys	52.6	55.6	
Girls	47.4	44.4	
BMI status at 12 months			<0.001
Normal BMI status	69.0	75.3	
High BMI status	31.0	24.7	

[†]Data are expressed as median and interquartile range (IQR) or %. Kruskal-Wills test and Chi-square test were used to compare continuous and categorical variables between groups. "P <0.05" is considered as significant difference.

[‡]Cohort A was conducted in Shenyang, Wuhan and Guangzhou cities (2009-2010). Cohort B was conducted in Zhuhai city (2014-2016)

Supplementary Table S4. The interactive effect between maternal pre-pregnancy BMI status and BP status during pregnancy on high BMI status in infants at 12 months old stratified by cohorts*

Pre-pregnancy BMI status‡	BP status during pregnancy‡	Model1† OR(95% CI)	*P*-value	Model2† OR(95% CI)	*P*-value	Model3† OR(95% CI)	*P*-value
**Cohort A**†							
Non-OWO‡	NBP‡	Reference		Reference		Reference	
Non-OWO‡	HDP‡	1.29(0.75, 2.22)	0.368	1.26(0.72, 2.19)	0.418	1.26(0.72, 2.21)	0.411
OWO‡	NBP‡	1.21(0.81, 1.80)	0.354	1.17(0.78, 1.76)	0.443	1.17(0.77, 1.76)	0.464
OWO‡	HDP‡	3.17(1.44, 6.96)	0.004	3.04(1.37, 6.74)	0.006	2.85(1.28, 6.34)	0.010
Multiplicative interaction							
OWO*HDP‡		2.04(0.73, 5.69)	0.171	2.06(0.73, 5.81)	0.171	1.94(0.69, 5.47)	0.213
Additive interaction							
OWO*HDP(RERI/AP)‡		1.68(-0.93,4.28)/ 0.53(0.08,0.98)		1.61(-0.92,4.14)/ 0.53(0.07,0.99)		1.42(-0.97,3.81)/ 0.50(0.01,0.99)	
**Cohort B**†							
Non-OWO‡	NBP‡	Reference		Reference		Reference	
Non-OWO‡	HDP‡	1.56(0.89, 2.74)	0.120	1.46(0.82,2.57)	0.197	1.42(0.80, 2.51)	0.233
OWO‡	NBP‡	1.28(0.89, 1.86)	0.189	1.16(0.79, 1.71)	0.439	1.16(0.79, 1.71)	0.436
OWO‡	HDP‡	3.85(1.17,12.68)	0.027	3.89(1.16, 13.07)	0.028	3.94(1.17, 13.23)	0.027
Multiplicative interaction							
OWO*HDP‡		1.92(0.49, 7.48)	0.346	2.30(0.58, 9.08)	0.235	2.39(0.60, 9.45)	0.215
Additive interaction							
OWO*HDP(RERI/AP)‡		2.00(-1.14,5.15)/ 0.52(0.07,0.97)		2.27(-0.92,5.46)/ 0.58(0.18,0.99)		2.36(-0.88,5.59)/ 0.60(0.21,0.99)	

† Logistic regression analysis. Model1: Unadjusted model; Model2: Adjusted for infant sex, gestational age, infant birth weight and length, delivery mode, maternal age, and maternal gestational weight gain; Model 3: Adjusted for potential variables in Model2 plus maternal education level, household income, secondhand smoke exposure, feeding patterns at 1 month. Cohort A was conducted in Shenyang, Wuhan and Guangzhou cities (2009-2010). Cohort B was conducted in Zhuhai city (2014-2016).

‡AP, attributable proportion; RERI, relative excess risk due to interaction; BMI: body mass index; BP: blood pressure; Non-OWO: non-overweight or obesity; OWO: overweight or obesity; NBP: normal blood pressure; HDP: hypertensive disorders of pregnancy.
